# High-protein intake and early exercise in adult intensive care patients: a prospective, randomized controlled trial to evaluate the impact on functional outcomes

**DOI:** 10.1186/s12871-021-01492-6

**Published:** 2021-11-13

**Authors:** José Raimundo Araujo de Azevedo, Hugo César Martins Lima, Pedro Henrique Dias Brasiliense Frota, Ivna Raquel Olimpio Moreira Nogueira, Suellen Christine de Souza, Erika Arana Arraes Fernandes, Adlyene Muniz Cruz

**Affiliations:** Intensive Care Unit, Hospital São Domingos, Av. Jerônimo de Albuquerque, 540 - Bequimão, São Luís, MA 65060-645 Brazil

**Keywords:** Protein, Resistance training, Critical care, Physical component summary, Indirect calorimetry, Outcome

## Abstract

**Background:**

We evaluated the efficacy of high protein intake and early exercise versus standard nutrition care and routine physiotherapy on the outcome of critically ill patients.

**Methods:**

We randomized mechanically ventilated patients expected to stay in the intensive care unit (ICU) for 4 days. We used indirect calorimetry to determine energy expenditure and guide caloric provision to the patients randomized to the high protein and early exercise (HPE) group and the control group. Protein intakes were 1.48 g/kg/day and 1.19 g/kg/day medians respectively; while the former was submitted to two daily sessions of cycle ergometry exercise, the latter received routine physiotherapy. We evaluated the primary outcome physical component summary (PCS) score at 3 and 6 months) and the secondary outcomes (handgrip strength at ICU discharge and ICU and hospital mortality).

**Results:**

We analyzed 181 patients in the HPE (87) and control (94) group. There was no significant difference between groups in relation to calories received. However, the amount of protein received by the HPE group was significantly higher than that received by the control group (*p* < 0.0001). The PCS score was significantly higher in the HPE group at 3 months (*p* = 0.01) and 6 months (p = 0.01). The mortality was expressively higher in the control group. We found an independent association between age and 3-month PCS and that between age and group and 6-month PCS.

**Conclusion:**

This study showed that a high-protein intake and resistance exercise improved the physical quality of life and survival of critically ill patients.

**Trial registration:**

Research Ethics Committee of Hospital São Domingos: Approval number 1.487.683, April 09, 2018. The study protocol was registered in ClinicalTrials.gov (NCT03469882, March 19,2018).

**Supplementary Information:**

The online version contains supplementary material available at 10.1186/s12871-021-01492-6.

## Background

Muscle weakness associated with critical illness has a significant impact on short- and long-term patient outcomes [1, 2]. Puthucheary et al. [3] analyzed 63 septic patients with imaging examination and established a clear relationship between the number of organ failures and muscle loss within the first 10 days of ICU admission. Although a study involving 244 critically ill patients showed an alarming relationship between reduced muscle mass at admission and mortality [4], there is unclear evidence that nutritional interventions can attenuate muscle loss and improve outcomes [5–7].

Concerning nutrition research in critically ill patients, an intensive care medicine research [8] agenda prioritized the evaluation of the effect of protein dose coupled with physical activity in the acute phase of critical illness. The optimal integration between adequate protein intake and exercise in critically ill patients may have an impact on short and long-term outcomes, but this hypothesis has not been tested in studies with good methodology. An ongoing randomized trial of combined cycle ergometry and amino acids supplementation in the ICU (NEXIS Trial) evaluates the effect of early bedside cycling and intravenous amino acids (to achieve a total protein intake of 2.0–2.5 g/kg/day) on the physical recovery of ICU patients assessed by the 6-min walk test [9].

In the given context, we conducted a prospective randomized controlled trial to evaluate the efficacy of high protein intake of 2.0 to 2.2 g/kg/day and early exercise versus recommended protein intake of 1.4 to 1.5 g/kg/day and routine physiotherapy on outcome of critically ill patients. As the primary outcome measure, we used the physical component summary (PCS) of the SF-36 quality of life instrument after 3 and 6 months of randomization. The secondary outcome measures comprised the ICU-acquired weakness through handgrip strength at the ICU discharge, the duration of mechanical ventilation, the ICU length of stay, and ICU and hospital mortality.

## Methods

This is a prospective, randomized, controlled trial conducted in a tertiary hospital’s clinical ICU (12 beds), surgical ICU (13 beds), and high complexity surgical and trauma ICU (12 beds). The trial only included patients aged above 18 years, who were admitted to ICU for more than 3 days, between June 2018 to June 2020, and were submitted to mechanical ventilation. Patients were excluded for the following reasons: if they were pregnant, moribund, under ventilation for more than 96 h before enrolment, and unable to walk without assistance before the acute illness that led to ICU admission (use of an assistance device to walk was not an exclusion criteria); if they suffered from severe cognitive impairment before hospitalization, neuromuscular diseases that compromised weaning from ventilation, acute pelvic fracture, unstable spinal trauma, and severe liver disease; if it was impossible to start a diet according to the institutional protocols; and if they did not sign the written informed consent. In some circumstances, patients were not included in the resistance exercise program because of temporary limiting factors such as the use of a neuromuscular blocking drug or a high dose of vasoactive drug, dependence on mechanical ventilation with FIO_2_ ≥ 60% and/or PEEP ≥12 cm H_2_O, intracranial hypertension, open abdomen, and uncontrolled status epilepticus.

For the patients who met the inclusion criteria, we collected the demographic data regarding the age, gender, the admission category (medical or surgical), the primary admission diagnosis, the simplified acute physiology score III (SAPS 3), admission sequential organ failure assessment (SOFA), and nutrition risk score (NRS-2002). We obtained written informed consent from the patients or their legal representatives. The trial protocol was approved by the Research Ethics Committee of the Hospital Sao Domingos Number 1.487.683 in April 09, 2018. The study protocol was registered in the ClinicalTrials.gov (NCT 03469882, March 19, 2018).

### Nutritional protocol

Patients were randomized in a 1:1 ratio to the high-protein and early exercise (HPE) group or the control group, using a table of random numbers and sealed envelopes. After randomization, on the third day, we initiated the nutrition therapy (preferably by the enteral route) and followed the ICU’s nutritional support protocol (Additional file 1). Patients who could not achieve the caloric goal after 7 days of the nutritional therapy were supported by complementary parenteral nutrition. Patients who developed high gastric residue (greater than 300 ml in 12 h) within the first 24 h of the enteral nutritional therapy received intravenous metoclopramide and erythromycin enterally. If the high residue persisted on the third day of the nutritional therapy, a post-pyloric nutrition catheter was inserted. Patients with absolute contraindications to enteral nutrition received parenteral nutrition. In both groups, the resting energy expenditure of the patients was measured daily by indirect calorimetry using the GE-Carescape B650 equipment (GE Healthcare Oy, Helsinki, Finland). On the third day, patients of both groups received 50% of the measured energetic expenditure (MEE) and 0,8–1,0 g/kg/day of protein. On the fifth day, determined by indirect calorimetry, the caloric intake was increased to 80% of the value and the protein intake was increased to reach the targeted 2.0–2.2 g/kg/day in the HPE group or 1.4–1.5 g/kg/day in the control group. We recorded daily data on the predicted and achieved caloric and protein intakes for 14 days or until the discharge or death.

The nutritional formula used in the HPE group was Peptamen Intense (1.0 kcal/ml, 93 g/l protein, Nestlé Healthcare). The control group received nutritional support according to the guidance of the attending physician and the nutrition team.

### Cycle ergometry exercise

Patients randomized to the HPE group were submitted to two daily 15-min sessions of cycle ergometry; the resistance of the cycle ergometer was increased gradually during the first week. These sessions were started immediately after the randomization and continued until the discharge, death, or 21 days of stay in the study (whichever comes first). We used a Moto Med Letto II cycle ergometer (ReckTechnik, Germany). In the control group, patients were submitted to the ICU’s physiotherapy protocol, which included early arrival of the bed and passive and active movements at least twice a day.

### Outcome measures

The primary outcome was the PCS score 3 and 6 months after randomization, which was obtained from the medical outcomes study 36-item short-form health survey (SF-36) [10, 11]. This tool was validated for the Brazilian population, and the responses were obtained through telephonic interview at 3 and 6 months after randomization. Patients that deceased before the primary endpoint received 0 points at the PCS score. Secondary outcomes included the evaluation of ICU-acquired weakness through handgrip strength (Saehan Hydraulic Hand Dynamometer, Saehan Corp, Korea), which was measured at ICU discharge or after 21 days of ICU stay. The ICU-acquired weakness was defined as handgrip strength, at < 11 kg-force for males and < 7 kg-force for females [8]. Secondary outcomes also included the duration of mechanical ventilation, the ICU length of stay, and ICU and hospital mortality.

Due to the nature of interventions, it was not possible to blind the study to clinicians caring for patients. To minimize bias blinded assessors performed all outcome assessments.

### Statistical analysis

The final sample comprised 180 patients. This number is necessary to detect a clinical difference of at least 5.5 points between the groups, in the PCS at 6 months, using an 80% power, with a significance level of 5% (*p* < 0.05). The calculation was based on a PCS score of 37.5 and a standard deviation of 10.65, according to the calculation used in the EAT-ICU study [12], initially comprising 60 patients per group. In line with a study with a power of around 50% [13], which was realized in this ICU, we considered the possible losses owing to the expected mortality rate. Given this, we increased the sample size to 60 patients, totaling 90 patients per group.

For the categorical variables, frequency and percentage were calculated and compared using the chi-square test. Numeric variables were expressed as median (interquartile range, IQR). The Shapiro-Wilk was applied for assessing the normality of the numeric variables.

In each group, the PCS 3 and PCS 6 were compared using the Mann-Whitney U test for the paired samples. Patients who died before 3 and 6 months were given the lowest possible PCS score (Zero). The Kaplan-Meier method was used to estimate the survival rate of patients in each group, and the log-rank test was used to compare the survival function between the groups.

The linear regression model, including the clinically relevant variables (age, gender, SAPS, SOFA, nutritional assessment, the nutrition risk score, and group), was adjusted to verify the influence of the intervention on the PCS score at 3 and 6 months. First, the univariate analysis was conducted; subsequently, the variables with *p*-value less than 0.20 were included in the multivariate analysis. Variables with a p-value of < 0.05 were considered statistically significant. Statistical analyses were computed in R 4.0.2 (R Core Team, 2017) [14].

## Results

**Fig. 1:**
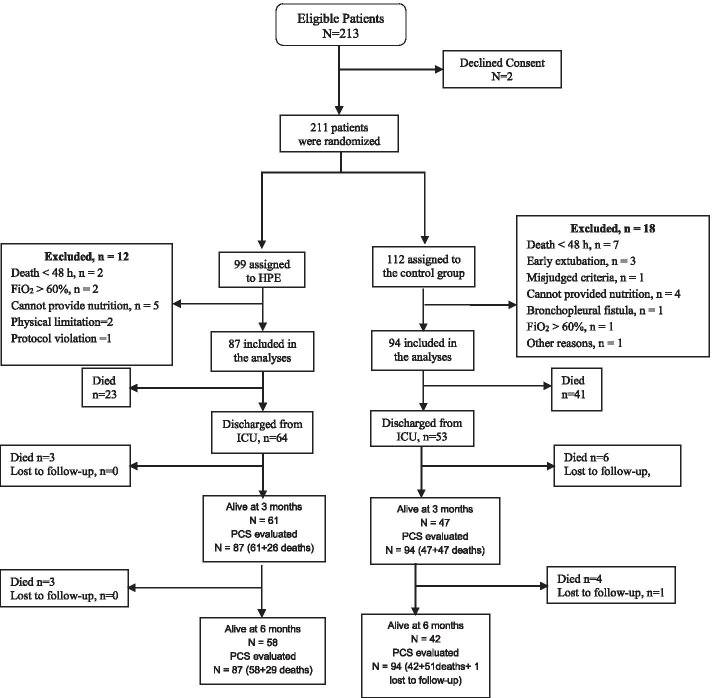
Flow diagram of study population

Between June 2018 and June 2020, 213 patients met the inclusion criteria, and 2 declined the consent to participate. We randomized the remaining 211 patients to the HPE group (99) and the control group (112). Post-randomization, based on the reasons explained in Fig. 1, we excluded 12 patients and 18 patients from the HPE and control groups, respectively. Thus, 181 patients were analyzed in the HPE group (87) and the control group (94). Table 1 shows that the demographic and clinical data were comparable between the two groups.

**Table 1:** Demographic and clinical characteristics of the study

Variable	HPE group *n* = 87	Control group *n* = 94	*P* value
Age (yr), mean (SD)	67.6 (17.8)	65.3 (19.7)	0,41
Female, n (%)	34 (39)	48 (51)	0,110
SAPS 3 score, Median (IQR)	55 (44–66)	50 (48.7–68)	0,07
SOFA score, Median (IQR)	5 (3–9)	6 (3.7–8)	0,73
NRS - 2002, Mean (SD)	4,1 (1,0)	4,2 (1,1)	0,59
Admission Category, n (%)			0,89
medical	64 (73)	70 (74)	
surgical	23 (27)	24 (26)	
Primary ICU diagnosis, n (%)			0,72
Respiratory	28 (32)	26 (30)	
Cardiovascular	6 (7)	8 (10)	
Neurological	17 (20)	17 (20)	
Gastrointestinal	9 (10)	8 (9)	
Renal	3 (4)	2 (3)	
Trauma	6 (7)	4 (5)	
Others	15 (17)	20 (23)	

SAPS 3, Simplified Acute Physiology Score; SOFA, Sequential Organ Failure Assessment; NRS-2002, Nutrition Risk Score-2002; IQR, Interquartile range

### Nutrition therapy

We did not notice any significant difference between the two groups in relation to the median (IQR) percentage of calories received, with the HPE and control groups at 81% (74.4–86.2) and 81.7% (74.0–90.2), *p* = 0.26, respectively. However, the median amount of protein received by the HPE group—1.48 g/kg/day (1.25–1.64)—was significantly higher than that received by the control group—1.19 g/kg/day (0.96–1.26), *p* < 0.0001. As determined by the protocol (Supplement 1) on the third day, patients received 50% of the measured energy expenditure (MEE); on the fifth day, both groups received 80% of the MEE and reached their protein goal. At this point, the HPE and control groups received 1.90 (1.7–2.1) g/kg/day and 1.34 (1.1–1.4) g/kg/day of protein (p < 0.0001), respectively (Table 2).

**Table 2:** Nutrition therapy

Variable	HPE group n = 87	Control group n = 94	*P* value
Measured energy Expenditure (MEE), kcal/day Median (IQR)	1.423 (1.239,8 - 1.731,5)	1.405 (1.206–1.620)	0.38
Pre-determined protein Requirement, g/kg/day	2,0 - 2,5	1.4–1.5	
Nutrition received			
- Calories (% MEE)			
Median (IQR)	81 (74.4–86.2)	81.7 (74.0–90.2)	0,26
- Total protein, g/kg/day			
Median (IQR)	1.48 (1.25–1.64)	1,19 (0.96–1.26)	< 0,0001
- Protein D3, g/kg/d,			
Median (IQR)	1.23 (0.85–1.60)	0,82 (0.66–1.19)	< 0,001
- Calories D3, kcal/kg/d,			
Median (IQR)	13.7 (11.3–17.0)	15 (12–18)	0,18
- Protein D7, g/kg/d,			
Median (IQR)	1.90 (1.7–2.1)	1.34 (1.10–1.45)	< 0,0001
- Calories D7, kcal/kg/d			
Median (IQR)	19.5 (16–22)	19.0 (14.3–21.4)	0,32

*MEE* measured energy expenditure, *IQR* interquartile range

### Physical component summary scores after 3 and 6 months

The PCS score was assessed 3 months in 87 (100%) and 94 (100%) patients, in the HPE and control groups. Of these, 26 and 47 patients in the HPE and control groups, respectively, died and received zero in the PCS score.

Six months after randomization, the PCS score was assessed in 87 (100%) and 93 (98,9%) patients in the HPE and control groups, respectively. Of these, 29 and 51 patients in the HPE and control groups, respectively, died and received zero in the PCS score. Table 3 shows that, at 3 months, the median (IQR) PCS score of 24.40 of the HPE group (0.00–49.12) was higher than that of the control group (0.00) (0.00–37.0), showing a statistical significance between the groups (*p* = 0.01). At 6 months, the PCS score of the HPE group (33.63) (0.00–71.61) was significantly higher than that of the control group (0.00) (0.00–55.1), with a statistical significance of *p* = 0.01.

**Table 3:** Primary and secondary outcomes

Variable	HPE group n = 87	Control group n = 94	*P* value
PCS score, Median (IQR)			
3 months	24.40 (0.00–49.12)	0.00 (0.00–37.0)	0,01
6 months	33.63 (0.00–71.61)	0.00 (0.00–55.1)	0,01
ICU-acquired weakness n (%)	16 (29.1)	26 (46.4%)	0.05
Length of stay, days			
Median (IQR)			
ICU	18 (12–36)	23 (16–36)	0,11
Hospital	38 (18–70)	40 (21–60)	0,96
Duration of MV, days			
Median (IQR)	10 (5–19)	12 (7–21)	0,09
Mortality			
n (%)			
ICU	23 (26.4)	41 (43.6)	0,01
Hospital	25 (31.2)	47 (53.4)	0,002
6-months follow-up	29 (33.3)	51 (54.2)	0.005

*PCS* physical component summary

In the logistic regression analysis of the PCS at 3 months, while the univariate analysis showed the statistical significance of age (*p* < 0.001), the body mass index (BMI) (*p* = 0.024), NRS-2002 (p < 0.001), and diagnostic category (p < 0.001), after adjusting for independent covariates, the multivariate analysis showed the statistical significance of age only (*p* < 0.001) (Table 4). Regarding the 6-month PCS, the univariate analysis showed the statistical significance of age (p < 0.001), NRS-2002 (p < 0.001), diagnostic category (*p* = 0.005), and group (p = 0,017); in the multivariate analysis, after adjusting for independent covariates, age (p < 0.001), NRS-2002 (p = 0,021), and group (p = 0,025) were significant (Table 5).

**Table 4:** Univariate and multivariate regression models of the risk factors associated with PCS after 3 months

Univariate				Multivariate		
Variable	Estimate	CI	P	Estimate	CI	p
Age	−0.88	−1.10–0.67	0.001	−0.73	−1.00–0.43	< 0.001
Male	8.49	−0.98–17.95	0.079	1.80	−6.70–10.30	0.677
BMI	0.91	0.12–1.17	0.024	−0.10	− 0.85–0.66	0.797
SAPS 3	−0.17	−0.44–0.09	0.189	0.06	−0.18–0.29	0.629
SOFA	0.59	−0.14-1.32	0.112	−0.08	−0.73–0.58	0.821
NRS-2002	−11.26	- 15.23–7.28	< 0.001	−3.98	−8.68–0.72	0.096
Diagnostic						
Category	15.74	5.15–26.33	0.004	6.26	−3.70–16.27	0.218
Protein	1.34	−10.2–12.94	0.821	–	–	–
Group	9.13	- 0.28–18.55	0.057	7.30	1.00–15.60	0.025

*CI* Confidence interval, *BMI* Body mass index, *SAPS 3* Simplified Acute Physiology Score, *SOFA*, Sequential organ failure assessment, *NRS* Nutritional risk screening

**Table 5:** Univariate and multivariate regression models of the risk factors associated with PCS after 6 months

Univariate				Multivariate		
Variable	Estimate	CI	p	Estimate	CI	p
Age	−0.97	−1.22–0.72	< 0.001	−0.73	−1.02–0.43	< 0.001
Male	8.11	−2.68–18.9	0.140	0.27	−9.33–9.87	0.956
BMI	1.20	0.30–2.09	0.009	0.02	−0.83–0.88	0.954
SAPS 3	−0.15	−0.45–0.15	0.320			
SOFA	0.63	- 0.20–1.45	0.137	−0.10	−0.84–0.63	0.784
NRS-2002	13.82	−18.27–9.36	< 0.001	−6.27	−11.59–0.96	0.021
Diagnostic						
Category	17.58	5.45–29.70	0.005	5.70	−5.51–16.90	0.317
Group	13.00	2.35–23.65	0.017	10.67	1.38–19.90	0.025

*CI* Confidence interval, *BMI* Body mass index, *SAPS 3* Simplified Acute Physiology Score, *SOFA*, Sequential organ failure assessment, *NRS* Nutritional risk screening

### Secondary outcomes

Handgrip strength was evaluated at the time of ICU discharge or after 21 days of ICU stay; it was used to determine the incidence of the ICU-acquired weakness. The measurement comprised 56 patients each in the HPE and control groups. The ICU-acquired weakness was identified in 16 (28,5%) and 26 (46.4%) patients in the HPE and control groups (*p* = 0.05). This borderline significance shows a trend that the ICU-acquired weakness was higher in the control group.

There was no difference between groups related to the ICU and hospital length of stay and the duration of mechanical ventilation. The ICU mortality rates in the HPE and control groups were 23 (26.4%) and 41 (43.6%) (*p* = 0.01), respectively. The hospital mortality rates in the HPE and control groups were 25 (31.2%) and 47 (53.4%) (*p* = 0.002), respectively and 6-months mortality were 29 (33.3%) in HPE group and 51 (54.2%) in control group (*p* = 0.005) (Table 3).

**Fig. 2:**
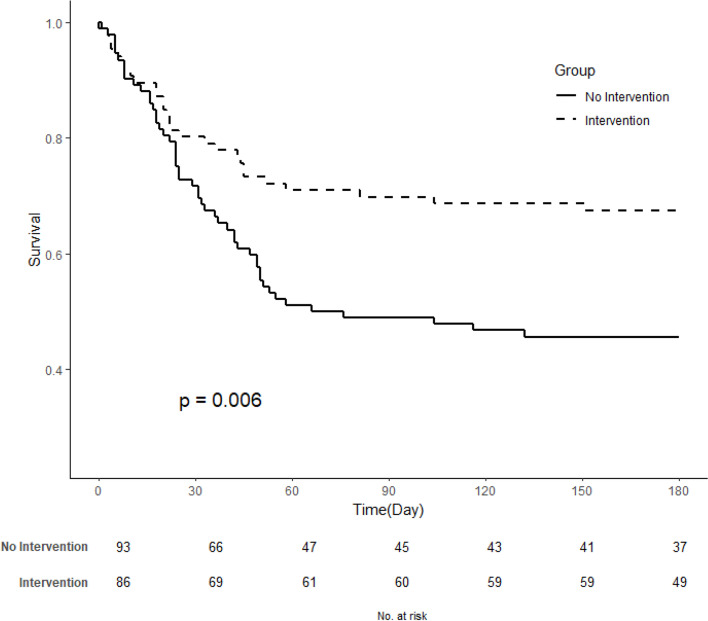
Survival curves of intervention and control groups

Figure 2 presents the Kaplan-Meier survival curves for the patients in the two groups. The curves show that the patients in the intervention group survived more than those in the control group. The difference between the two survival curves was statistically significant (*p* = 0.006).

## Discussion

In this prospective randomized controlled trial of mechanically ventilated patients admitted to the ICU for at least 4 days, we found a significant improvement in the PCS score assessed at 3 and 6 months in the patients of the HPE group. We also found a significant reduction in mortality in the HPE group. The multivariate analysis showed an independent association between lower age and a better 3-month PCS and that between a better 6-month PCS and lower age, lower nutrition risk, and belonging to the HPE group. In the HPE group, we observed a borderline improvement in the acquired weakness evaluated through handgrip strength at the time of ICU discharge or after 21 days of ICU stay. To the best of our knowledge, this is the first study to demonstrate that a high protein intake coupled with early resistance training improves the physical component of quality of life and, more importantly, the mortality in critically ill patients. In a previous study [13], we compared critically ill patients that received a high protein intake with those that received routine protein intake and demonstrated that receiving less than the predicted protein target was associated with a lower PCS score at 3 and 6 months.

Allingstrup et al. [12] analyzed 199 patients randomized to receive a caloric intake determined by indirect calorimetry; they also received a higher protein intake than a group receiving the usual protein intake and 25 kcal/kg/day. The study found no difference in the PCS score for quality of life between the two groups, when assessed 6 months after randomization. There are significant differences between our study and that of Allingstrup et al. In that study, urinary urea was used to determine the protein supply in the study group, but the patients received 1.5 g / kg of protein from the first day as well as the energy supply was 100% of what was measured by calorimetry. Ferrie et al. [15] randomized ICU patients to receive parenteral nutrition at 1.2 g / kg / day of protein, when compared with 0.8 g / kg / day; the authors did find differences in short-term outcomes but no difference in long-term outcomes. In a cohort of 726 non-septic ICU patients, Weijs et al. [16] found that the mortality declined with a high protein intake but increased with energy overfeeding. Nicolo et al. [17] analyzed 2824 critically ill patients who remained in the ICU for at least 4 days; the study evaluated the impact of protein delivery on mortality and observed that the administration of the goal protein higher than that of 80% led to a 40% reduction in mortality. Conversely, an increase in energy delivery was not associated with a reduction in mortality. Looijaard et al. [18], in a recent study, evaluated patients with a low skeletal muscle area and density on admission; the findings revealed that early protein intake ≥1.2 g/kg/d was associated with a lower mortality in patients with a low skeletal muscle area and density.

Despite the aforementioned evidence, there is ambiguity regarding the ideal protein intake and the influence of the interrelationship between the caloric and protein doses offered to critically ill patients. It must also be noted that the optimal timing for protein delivery [8] is necessary to identify the subgroups that may specifically benefit from early protein intake [19, 20]. Based on the nutritional protocol adopted for this study, we gradually increased the protein intake in the first week, with a low protein intake during the first days and a higher protein intake from the fifth day. This protocol is in line with some studies that suggested a time-dependent association of protein intake in the first days and an association with the clinical outcome. Casaer et al. [5] suggested that a higher intake of protein in the first 3 days might be harmful. Koekkoek et al. [21] suggested that a gradual increase in the protein intake during the first week is associated with a lower 6-month mortality. In our protocol, the protein intake was initiated on the third day; in both groups, patients received 0,8 to 1,0 g/kg/day of protein, and the caloric intake was determined by indirect calorimetry.

Evidence that a considerable loss in muscle mass and ICU-acquired weakness, in critically ill patients, impacts outcomes has stimulated initiatives for early physical rehabilitation. Studies have shown a positive relationship between early rehabilitation and outcomes. For example, findings showed better functional capacity and shorter hospital length of stay [4, 22] and better functional capacity after 6 months of hospital discharge, when evaluated by the SF-36 [23, 24], with early rehabilitation than standard care.

Although studies have recognized the influence of early physical rehabilitation on outcomes and the important role of the optimization of protein intake in these patients, these findings have not been discussed in relation to critically ill patients. In our study, in the HPE group, the patients received a high protein intake and resistance training twice daily. They showed an expressive reduction in mortality, a borderline advantage in acquired weakness, and a better PCS at 3 and 6 months than that of the control group. Although the significant reduction in mortality in the HPE group was somewhat surprising, several observational studies had already revealed a significant reduction in mortality in patients who received a high protein intake [16–18]. We understand that in this study, the reduction in mortality can be explained by a set of interventions. They are: progressive increase in protein intake, avoiding the adverse effects of an early high protein intake [5, 21]; high protein intake from the fifth day of treatment^,18^ and early physical rehabilitation [4, 22–24].

Concerning the strengths of our trial, the randomized design lowers the risk of bias; the strength also lies in the blinded outcome assessment of the PCS score. Our trial succeeded in providing the nutrition according to the previously defined goals, wherein both the groups received different amounts of protein enterally and reached the goals of our protocol. We had a minimum loss during our 6-month follow-up in both groups.

Our study also has limitations. Our trial is a single-center trial made in three ICUs. The nutritional protocol was not blinded to the staff, which may possibly introduce bias. Even though we used indirect calorimetry for all the patients, only a few measures were reported for some of the patients because of mechanical ventilation limitations (high FiO_2_ or PEEP) or fast weaning of the ventilation. Although 30 patients were excluded from the study after randomization, it was not possible to perform an intention-to-treat analysis because these patients were excluded from the study within the first 48 h after randomization, that is, before the interventions were carried out. It was infeasible to measure the handgrip strength for all the patients. After discharge from the ICU, most patients received an oral diet and the physiotherapy conduct was determined by the ward team.

## Conclusion

In this prospective randomized controlled trial, we found that a high protein intake and resistance training led to an improvement in the physical quality of life of critically ill patients as measured by the PCS score after 3 and 6 months. We also found a reduction in mortality rate and a tendency to improvement in in the ICU-acquired weakness measured through handgrip strength in the study group. Although our findings are promising, further multicentric and randomized controlled trials are necessary.

## Supplementary Information

**Additional file 1.** Nutritional Protocol in HSD ICU.

**Additional file 2: Table S1**: Nutritional Protocol in HSD ICU.

## Data Availability

The dataset used and/or analyzed during the current study are available from the corresponding author on reasonable request.

## Abbreviations

ICU: Intensive Care Unit
HPE group: High protein and early exercise group
PCS: Physical component summary
SF-36: Short form survey for quality of life
SAPS 3: Simplified acute physiology score version 3
SOFA: Sequential organ failure assessment
MEE: Measured energy expenditure
