# Supplementary material for: High-protein intake and early exercise in adult intensive care patients: a prospective, randomized controlled trial to evaluate the impact on functional outcomes
Source: BMC Anesthesiol. 2021 Nov 13;21:283. doi: 10.1186/s12871-021-01492-6 (PMC8590269; doi:10.1186/s12871-021-01492-6)
Supplement: Supplementary file 1 — Additional file 1. Nutritional Protocol in HSD ICU. [file 12871_2021_1492_MOESM1_ESM.docx]

**Additional file 1. Nutritional Protocol in HSD ICU**

Our protocol comprised 5 steps, according to the days of the nutritional therapy. Day 1 – Fasting; Day 2 – Initiation of the supplementation with micronutrients (trace elements, thiamine, vitamin C, and a pack of multivitamins); Days 3 and 4 –Nutritional therapy with 50% to 70% of the resting energy expenditure (REE) measured by indirect calorimetry and 0.8 to 1.0 g/kg/day of protein; Days 5 and 6 – Increase in the caloric intake to 80% of REE measured by IC, and protein intake of 2.0 to 2.5 g/kg/day and 1.4 to 1.5 grams/kg/day, respectively, in the HPE and control groups; and Days 7 to 10 – If protein intake goal was not reached, consider starting supplementary parenteral nutrition.
