# Supplementary material for: High-protein intake and early exercise in adult intensive care patients: a prospective, randomized controlled trial to evaluate the impact on functional outcomes
Source: BMC Anesthesiol. 2021 Nov 13;21:283. doi: 10.1186/s12871-021-01492-6 (PMC8590269; doi:10.1186/s12871-021-01492-6)
Supplement: Supplementary file 2 — Additional file 2: Table S1: Nutritional Protocol in HSD ICU. [file 12871_2021_1492_MOESM2_ESM.docx]

**Table S1: Nutritional Protocol in HSD ICU**

| **Days** | **Nutrition Protocol** |
| --- | --- |
| Day 1 | - Fasting |
| Day 2 | - Beginning of the supplementation with micronutrients (trace elements, thiamine, vitamin C, and a pack of vitamins (Cerne)) |
| Days 3 and 4 | - Nutritional therapy with 50% to 70% of the resting energy expenditure measured by indirect calorimetry - 0.8 to 1.0 g/kg/day of protein - Maintenance of trace elements, thiamine, vitamin C, and a pack of vitamins |
| Day 5 | - Increase in the caloric intake to 80% of REE measured by calorimetry. - Protein intake of 2.0 to 2.2 g/kg/day in HPE group and 1.4 to 1.5 g/kg/day in the control group - Maintenance of trace elements, thiamine, vitamin C, and a pack of vitamins until day 6 |
| Day 7 to 10 | - If protein intake goal was not reached, consider start parenteral nutrition |
